# Cardiac diastolic function after recovery from pre-eclampsia

**DOI:** 10.5830/CVJA-2017-031

**Published:** 2018

**Authors:** Soma-Pillay P, Adeyemo AO, Louw MC, Adeyemo AO, Soma-Pillay P, Makin J, Pattinson RC

**Affiliations:** Cardiac Obstetric Unit, Department of Obstetrics and Gynaecology, University of Pretoria, Steve Biko Academic Hospital, Pretoria, South Africa; Cardiac Obstetric Unit, Department of Obstetrics and Gynaecology, University of Pretoria, Steve Biko Academic Hospital, Pretoria, South Africa; Department of Cardiology, University of Pretoria, Steve Biko Academic Hospital, Pretoria, South Africa; MediClinic Heart Hospital, Pretoria, South Africa; South African Medical Research Council Maternal and Infant Health Care Strategies Unit, Department of Obstetrics and Gynaecology, University of Pretoria, Pretoria, South Africa; South African Medical Research Council Maternal and Infant Health Care Strategies Unit, Department of Obstetrics and Gynaecology, University of Pretoria, Pretoria, South Africa; South African Medical Research Council Maternal and Infant Health Care Strategies Unit, Department of Obstetrics and Gynaecology, University of Pretoria, Pretoria, South Africa

**Keywords:** pre-eclampsia, diastolic function, left ventricular remodelling, pregnancy

## Abstract

**Background:**

Pre-eclampsia is associated with significant changes to the cardiovascular system during pregnancy. Eccentric and concentric remodelling of the left ventricle occurs, resulting in impaired contractility and diastolic dysfunction. It is unclear whether these structural and functional changes resolve completely after delivery.

**Aims:**

The objective of the study was to determine cardiac diastolic function at delivery and one year post-partum in women with severe pre-eclampsia, and to determine possible future cardiovascular risk.

**Methods:**

This was a descriptive study performed at Steve Biko Academic Hospital, a tertiary referral hospital in Pretoria, South Africa. Ninety-six women with severe preeclampsia and 45 normotensive women with uncomplicated pregnancies were recruited during the delivery admission. Seventy-four (77.1%) women in the pre-eclamptic group were classified as a maternal near miss. Transthoracic Doppler echocardiography was performed at delivery and one year post-partum.

**Results:**

At one year post-partum, women with pre-eclampsia had a higher diastolic blood pressure (p = 0.001) and body mass index (p = 0.02) than women in the normotensive control group. Women with early onset pre-eclampsia requiring delivery prior to 34 weeks’ gestation had an increased risk of diastolic dysfunction at one year post-partum (RR 3.41, 95% CI: 1.11–10.5, p = 0.04) and this was irrespective of whether the patient had chronic hypertension or not.

**Conclusion:**

Women who develop early-onset pre-eclampsia requiring delivery before 34 weeks are at a significant risk of developing cardiac diastolic dysfunction one year after delivery compared to normotensive women with a history of a low-risk pregnancy.

Pre-eclampsia is a pregnancy-specific disorder characterised by new-onset hypertension and proteinuria after 20 weeks’ gestation. Hypertensive disorders in pregnancy have been one of the top five causes of maternal mortality in South Africa for more than a decade.^1^ It was previously believed that the complications of pre-eclampsia ended with the delivery of the foetus and placenta, however it is now well established that pre-eclampsia is a risk for future hypertension, ischaemic heart disease, stroke and venous thromboembolism.^2^

Pregnancy is associated with significant haemodynamic and hormonal changes affecting the cardiovascular system. There is a 20% increase in cardiac output by eight weeks’ gestation.^3^ Peripheral vasodilatation leads to a 20 to 30% fall in systemic vascular resistance and a 40% increase in cardiac output. The heart undergoes remodelling, with an increase in left ventricular wall thickness and mass.^4^

Despite these changes, the left ventricular contractile function is maintained and any changes in cardiac geometry are rapidly reversible within three months post-partum in normotensive women.^4^ By contrast, vascular reactivity is augmented in pregnancies affected by pre-eclampsia.^5^ Pre-eclampsia results in a state of increased vascular stiffness, generalised vasoconstriction and a high total vascular resistance and low cardiac output compared to the changes seen in a normal pregnancy.^5^

Cardiac changes classically associated with pre-eclampsia are diastolic dysfunction and an after-load-mediated left ventricular remodelling of the maternal heart.^6^-^8^ The heart remodelling is a response to the increased systemic afterload in order to minimise myocardial oxygen demand and preserve left ventricular function.

About 20% of women with pre-term pre-eclampsia and severe disease undergo severe left ventricular hypertrophy with advanced cardiac dysfunction.^9^ Typically there is preservation of both left atrial geometry and function, and left ventricular systolic function.^4^,^10^ The right ventricle is also usually unaffected.^10^ Levels of brain naturetic peptide (BNP) increase in pregnancies complicated by pre-eclampsia, and Fayers et al. have shown that the increase in BNP is accompanied by changes in left ventricular diastolic function.^11^ Elevated BNP levels are possibly the result of myocardial remodelling and sub-clinical ventricular dysfunction that accompanies the severe vasoconstriction observed in pre-eclampsia.^11^

Diastolic dysfunction is described as impaired left ventricular filling and may be present in the setting of normal or abnormal systolic function. Pre-clinical diastolic dysfunction is associated with the development of future heart failure and is a predictor of all-cause mortality.^12^ Diastolic filling abnormalities may also play a significant role in the pathogenesis of pulmonary oedema, complicating hypertensive crises in pregnancy.^13^

Desai et al. found that diastolic filling abnormalities were demonstrated in a significant proportion of pre-eclamptic pregnancies complicated by pulmonary oedema compared to control groups of women who were hypertensive and normotensive in pregnancy.13 The authors of this study postulated that the diastolic filling abnormalities demonstrated in the study occurred within a short time frame of severe pre-eclampsia in pregnancy or could represent pre-eclampsia superimposed on established hypertension.

Whether diastolic dysfunction persists after delivery is uncertain. Identifying factors that may affect future cardiovascular risk may identify a group of women requiring increased postpartum vigilance and lifestyle modification. The aim of this study was to determine cardiac diastolic function at delivery and one year post-partum in women with severe pre-eclampsia and to further determine possible future cardiovascular risk.

## Methods

This was a descriptive study of women with severe pre-eclampsia, performed at Steve Biko Academic Hospital from 1 April 2013 to 30 March 2016. The Cardiology Department at Steve Biko Academic Hospital reserved echocardiographic appointments every Wednesday during the study period. Post-partum women with severe pre-eclampsia were identified on a Wednesday morning and if fit to be transported to the cardiology clinic, were informed of the study. Echocardiographic studies were performed on patients who consented to the procedure and were agreeable to follow-up studies.

One hundred and six women with severe pre-eclampsia and 45 normotensive, low-risk women who served as the control group were identified and recruited shortly after delivery. Women with structural heart disease or pulmonary embolus were excluded from the study. Women diagnosed with maternal metabolic syndrome were not recruited to the control group.

Echocardiograms of the maternal heart were performed between day two and seven post-delivery and follow-up scans were done after one year. Hypertensive disorders were classified according to the classification and diagnosis of the International Society for the Study of Hypertension in Pregnancy (ISSHP).^14^

Doppler echocardiography was carried out by the Department of Cardiology at Steve Biko Academic Hospital. The following echocardiographic parameters were assessed in the evaluation of diastolic dysfunction: left ventricular ejection fraction (LVEF), mitral E-wave (E) and mitral A-wave velocities (A), E/A ratio, mitral E-velocity deceleration time (DT), lateral early diastolic (e′) velocity tissue Doppler and E/e′ ratio.

The diagnosis of diastolic dysfunction was made by a clinician in the cardiac-obstetric unit. All women diagnosed with diastolic dysfunction had the following minimum positive criteria: average E/e′ > 14 and lateral e′ velocity < 10 cm/s. The American Society of Echocardiography and the European Association of Cardiovascular Imaging have described the advantages and limitations used to assess left ventricular diastolic function15 (Table 1)

**Table 1:** Utility, advantages and limitations of variables used to assess left ventricular diastolic function15 (reproduced with permission)

*Variable*	*Physiological background*	*Advantages*	*Limitations*
Mitral E velocity	Reflects the LA–LV pressure gradient during early diastole and is affected by alterations in the rate of LV relaxation and LAP	Feasible and reproducible	Directly affected by alterations in LV volumes and elastic recoil. Age dependent
Mitral A velocity	Reflects the LA–LV pressure gradient during late diastole, which is affected by LV compliance and LA contractile function	Feasible and reproducible	Sinus tachycardia, first-degree AV block and paced rhythm can result in fusion of the E and A waves. If mitral flow velocity at the start of the atrial contraction is > 20 cm/s, A velocity may be increased. Age dependent
Mitral E/A ratio	Mitral inflow E/A ratio and DT are used to identify the filling patterns	Feasible and reproducible. Provides diagnostic and prognostic information. A restrictive filling pattern in combination with LA dilatation in patients with normal EFs is associated with a poor prognosis similar to a restrictive pattern in dilated cardiomyopathy	The U-shaped relationship with LV diastolic function makes it difficult to differentiate normal from pseudonormal filling, particularly with normal LVEF, without additional variables. If mitral flow velocity at the start of atrial contraction is > 20 cm/s, E/A ratio will be reduced due to fusion. Age dependent
Mitral E-velocity DT	DT is influenced by LV relaxation, LV diastolic pressures following mitral valve opening, and LV stiffness	Feasible and reproducible. A short DT in patients with reduced LVEF indicates increased LVEDP with high accuracy both in sinus rhythm and in AF	DT does not relate to LVEDP in normal LVEF. Should not be measured with E and A fusion due to potential inaccuracy. Age dependent
Pulsed-wave TDI-derived mitral annular early diastolic velocity: e′	A significant association is present between e′ and the time constant of LV relaxation shown in both animals and humans The haemodynamic determinants of e′ velocity include LV relaxation, restoring forces and filling pressure	Feasible and reproducible. LV filling pressures have a minimal effect on e′ in the presence of impaired LV relaxation. Less load dependent than conventional bloodpool Doppler parameters	Need to sample at least two sites with precise location and adequate size of sample volume. Different cut-off values depending on the sampling site for measurement. Age dependent
Mitral E/e′ ratio	e′ velocity can be used to correct for the effect of LV relaxation on mitral E velocity, and E/e′ ratio can be used to predict LV filling pressures	Feasible and reproducible. Values for average E/e’ ratio < 8 usually indicate normal LV filling pressures, values > 14 have high specificity for increased LV filling Pressures	E/e′ ratio is not accurate in normal subjects, patients with heavy annular calcification, mitral valve and pericardial disease. ‘Gray zone’ of values in which LV filling pressures are indeterminate. Different cut-off values depending on the sampling site for measurement

LV, left ventricular; LA, left atrial; LAP, left atrial pressure; LVEF, left ventricular ejection fraction; DT, mitral E-velocity deceleration time; e′, lateral early diastolic velocity; AF, atrial fibrillation.

Descriptive statistics in the form of means and standard deviations in the case of continuous data, and frequencies and percentages in the case of categorical data were calculated. A p-value of < 0.05 was considered significant. Ethical approval for the study was obtained from the University of Pretoria Ethics Committee (No. 125/2013).

## Results

There were 6 536 deliveries at our hospital during the recruitment phase of the study (1 April 2013 – 30 March 2015). Four hundred and sixty-three (7.1%) women presented with severe pre-eclampsia and 106 women were recruited to the study. Ten women were lost to follow up. Data were therefore recorded for 96 women with severe pre-eclampsia and 45 controls.

Seventy-four (77.1%) women in the study group for whom data were available fulfilled the World Health Organisation (WHO) criteria for the classification of a maternal near miss. Of the 96 women with severe pre-eclampsia, 14 were diagnosed with chronic hypertension and four with diabetes prior to pregnancy. At one year, the mean diastolic blood pressure and mean body mass index was significantly higher among the women who had pre-sclampsia during pregnancy compared to the normotensive control group. Table 2 describes the demographic data of the study population.

**Table 2:** Demographic data of the study population

*Characteristics*	*Pre-eclamptic group (n = 96)*	*Control group (n = 45)*	*p-value*
Age, years
Mean (SD)	28.9 (6.83)	27.2 (7.14)	0.66
Range	18–46	20–42
Race
African, n (%)	86 (89.58)	38 (84.44)
Caucasian, n (%)	5 (5.20)	3 (6.67)
Coloured, n (%)	4 (4.17)	4 (8.89)
Indian, n (%)	1 (1.04)	0 (0)
Obstetric history
Parity mean (range)	1.3 (0–4)	1.6 (0–5)
Timing of delivery
< 34 weeks, n (%)	44 (45.83)	0 (0)
34–37 weeks, n (%)	25 (26.04)	5 (11.11)
> 37 weeks, n (%)	27 (28.13)	40 (88.89)
Medical conditions
Diabetic at 1 year, n (%)	6 (6.25)	0 (0)	
Hypertensive at 1 year, n (%)	52 (54.17)	2 (4.44)	
Haemoglobin at 1 year (g/dl)			
Mean (SD)	12.02 (1.46)	12.42 (1.13)	0.15
Blood pressure at 1 year (mmHg)			
Systolic, mean (SD)	128.01 (14.17)	115.08 (9.89)	0.08
Diastolic, mean (SD)	80.91 (14.47)	72.45 (9.16)	0.001
BMI at 1 year, mean (SD)	30.27 (7.55)	28.04 (3.64)	0.02

Twenty women (20.83%) with pre-eclampsia were diagnosed with diastolic dysfunction at delivery compared with six (13.3%) of the controls (p = 0.26). Of the 20 women who were diagnosed with diastolic dysfunction at delivery, 13 (65%) had early-onset pre-eclampsia, requiring delivery prior to 34 weeks. At one year, 11 (11.46%) women with pre-eclampsia were diagnosed with diastolic dysfunction compared with three (6.67%) in the control group (RR = 1.67; p = 0.27).

**Fig. 1:**
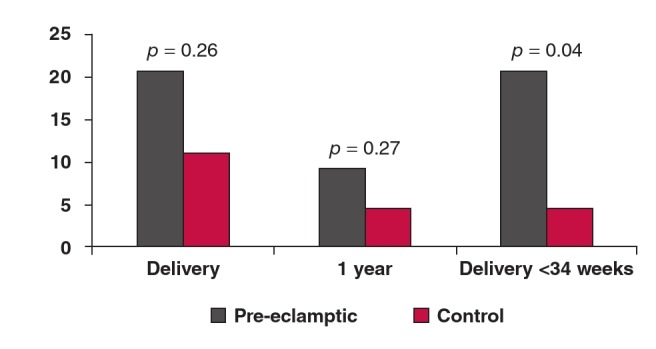
Risk of diastolic dysfunction at delivery and at one year, and at one year for sub-group of women with early-onset pre-eclampsia requiring delivery prior to 34 weeks.

Women with early-onset pre-eclampsia requiring delivery prior to 34 weeks’ gestation had an increased risk of diastolic dysfunction at one year post-partum (RR 3.41, 95% CI: 1.11– 10.5, p = 0.04) (Fig. 1). Delivery prior to 34 weeks was associated with an increased risk of diastolic dysfunction even if patients with chronic hypertension at one year were excluded from the analysis (p = 0.02, 95% CI: 1.43–97.67) There was no significant association between diastolic dysfunction and chronic hypertension at one year (RR = 2.02, p = 0.33, 95% CI: 0.57– 7.13). Echocardiographic measurements of diastolic function after one year are shown in Table 3.

**Table 3:** Cardiac diastolic function at one year

	*Pre-eclamptic group, mean (SD)*	*Control group, mean (SD)*	*p-value*
Left ventricular ejection fraction, %	60.54 (7.62)	63.43 (4.88)	0.08
E velocity, m/s	0.98 (0.20)	0.95 (0.14)	0.90
A velocity, m/s	0.70 (0.24)	0.64 (0.05)	0.01
E/A ratio	1.42 (0.39)	1.46 (0.12)	0.74
E-deceleration time (ms)	224.57 (51.00)	225.43 (35.09)	0.08
Lateral e′ (cm/s)	10.83 (2.86)	11.80 (1.99)	0.02
E/e′ ratio	10.11 (5.32)	9.96 (2.25)	0.11

Left ventricular systolic function was normal and similar in both groups, suggesting preservation of systolic function in both pre-eclamptics and controls. There was a significant decrease in lateral e′ and a significant increase in A velocity between the pre-eclamptic and control group at one year.

## Discussion

Heart failure is a progressive condition, which begins with risk factors for left ventricular dysfunction and progresses further to asymptomatic changes in cardiac structure and function, finally evolving into heart failure.^16^ Myocardial remodelling starts before the onset of symptoms. Diastolic dysfunction precedes the onset of systolic dysfunction in 50% of cardiac diseases, which further precedes the onset of heart failure.^5^

The American College of Cardiology has highlighted the importance of identifying asymptomatic cardiac dysfunction for early intervention and improvement of outcome.^17^ The risk for left ventricular diastolic dysfunction is significantly associated with higher age, body mass index (BMI), heart rate and systolic blood pressure.^16^ The prevalence of diastolic dysfunction in a general population aged less than 49 years was found to be 6.8%, and 27.3% for the total population, which included study subjects older than 70 years.^16^

Zanstra et al. found that 24% of women with the metabolic syndrome during pregnancy had diastolic dysfunction at six months post-partum, compared to 6.3% of women with low-risk pregnancies.^18^ Obesity and diastolic hypertension were strong correlates to diastolic dysfunction.

The rate of diastolic dysfunction at one year in the two groups of women with early-onset pre-eclampsia (22.7%) and low-risk pregnancies (6.7%) in our study were similar to rates reported by Zanstra et al.^16^ Although our study did not find associations between diastolic blood pressure and obesity with diastolic dysfunction, women in the pre-eclamptic group had a significantly higher BMI and diastolic blood pressure than those in the control group. Additionally, diastolic dysfunction is also a risk factor for future death.

The Olmsted study described the predictive significance of left ventricular diastolic dysfunction using multivariableadjusted analyses.^19^ The hazard ratio for all-cause mortality was 8.31 (p < 0.001) for mild diastolic dysfunction and 10.17 for moderate to severe diastolic dysfunction (p < 0.001). At one year post-delivery, diastolic dysfunction was present in 11.5% of women with pre-eclampsia, in 22.7% of women with early-onset pre-eclampsia and in 1.9% of women whose pre-eclampsia developed after 34 weeks. Women with early-onset pre-eclampsia requiring delivery prior to 34 weeks, irrespective of the presence of chronic hypertension, were at risk of developing diastolic dysfunction at one year post-delivery. Chronic hypertension, therefore, was not an additional risk factor for diastolic dysfunction at one year in women with earlyonset pre-eclampsia.

This study found that early-onset pre-eclampsia was a risk factor for diastolic dysfunction, while women who developed pre-eclampsia after 34 weeks had a risk similar to that of low-risk parous women (RR 3.41, 95% CI: 1.11–10.5, p = 0.04). This may be explained by the proposed differences in pathophysiology between early- and late-onset pre-eclampsia.

Redman et al. have suggested that pre-eclampsia could be the result of intrinsic or extrinsic placental causes.^20^ In early-onset pre-eclampsia, factors extrinsic to the placenta affect the uteroplacental circulation via incomplete spiral artery remodelling, while in late-onset disease, intrinsic factors affect the size of the placenta, restricting intervillous perfusion.20

The placentas of women with early-onset disease differ significantly from those who develop pre-eclampsia at term.^21^ The former group demonstrate placental findings consistent with insufficiency and vascular lesions, while late-onset disease is characterised by placental hyperplasia and unimpaired foetal growth.^21^-^24^ Further evidence suggesting that pre-eclampsia is more than one disease comes from differences in biochemical markers, Doppler studies and clinical features of the disease.^25^-^30^

Pre-eclampsia is a known risk factor for future chronic hypertension. Hypertension and hypertensive heart disease are one of the key contributors to the burden of non-communicable cardiovascular disease in Africa. Young African women are bearing the brunt of this increasing public health problem.^31^,^32^ Several studies have found that women from sub-Saharan Africa have the greatest risk of developing pre-eclampsia and eclampsia.^33^,^34^

Nakimuli et al., in a study of pre-eclampsia in women of African ancestry, found that African ancestry was the second strongest risk factor for pre-eclampsia after chronic hypertension.35 African ancestry was also a risk factor for earlyonset pre-eclampsia and poor obstetric outcomes such as foetal growth restriction and stillbirth.^35^ Pregnancy-related deaths from pre-eclampsia are also three times higher in women of African ancestry compared with Europeans.36 Almost 90% of women in our study were of African origin.

It is estimated that for every woman who dies during pregnancy or childbirth, 20 others will suffer severe morbidity.^37^ Most maternal mortality and morbidity datasets record information for up to 42 days post-partum. However women who develop pre-eclampsia during pregnancy, especially those with early-onset disease, may develop heart failure several years after pregnancy, resulting in the problem not being adequately identified and addressed.

The prognosis of women with compromised cardiac function is poorer than that of men.18 Women often present with atypical symptoms, resulting in delayed presentation, delayed diagnosis and suboptimal care compared to men.^38^,^39^ These factors highlight the need to identify women at risk of future cardiovascular disease, with the aim of reducing potential modifiable risk factors. Blood pressure control, weight loss and a low-sodium diet are important measures that have been identified with favourable changes in ventricular diastolic function.18 The American Heart Association Guideline on Lifestyle Management to reduce cardiovascular risk for adults who would benefit from blood pressure lowering include dietary modification appropriate to calorie requirements, reduction in salt intake and three to four sessions of aerobic activity per week lasting on average 40 minutes per session.^40^

This is the first study to evaluate diastolic function in a pre-eclamptic group of predominantly African population. Although we did not look at other risk factors for cardiovascular disease in this population, the study provides valuable information in identifying a potential group of women at risk of disease at an early stage. This would provide opportunities for screening and lifestyle modification.

The strength of this study is that it is one of the first to look at cardiac diastolic function in an African population where the rates of hypertension both during and outside of pregnancy were high. A possible limitation is that most patients were seen for the first time during pregnancy, with severe acute hypertension. Only 14.6% of women were known to have chronic hypertension. It is possible that some women had undiagnosed chronic hypertension – this is especially likely as the rate of chronic hypertension postpartum at one year was 54.2%. Some of the women with undiagnosed chronic hypertension may have had pre-existing diastolic dysfunction that could have been worsened by the superimposed pre-eclampsia. A further limitation is that only a select group of pre-eclamptic women were recruited to the study.

## Conclusion

Women who develop early-onset pre-eclampsia requiring delivery prior to 34 weeks’ gestation have an increased risk of cardiac diastolic dysfunction one year after delivery. Diastolic dysfunction precedes the onset of systolic dysfunction and clinical heart failure. A strategy to screen and treat women with cardiovascular risk, particularly in lower- and middle-income countries should be explored further.
